# Agonistic behaviour in juvenile southern rock lobster, *Jasus edwardsii* (Decapoda, Palinuridae): implications for developing aquaculture

**DOI:** 10.3897/zookeys.457.6760

**Published:** 2014-11-25

**Authors:** Chris G. Carter, Heath Westbury, Bradley Crear, Cedric Simon, Craig Thomas

**Affiliations:** 1 Institute for Marine and Antarctic Studies, University of Tasmania, Australia

**Keywords:** Spiny lobster, aquaculture, con-specific, behaviour, inter-individual interaction, growth depensation

## Abstract

The Southern rock lobster, *Jasus edwardsii*, is a temperate species of spiny lobster with established well managed fisheries in Australia and New Zealand. It has also been under consideration as a species with aquaculture potential. Agonistic behaviour has important consequences under aquaculture conditions that encompass direct effects, such as damage or death of protagonists, and indirect effects on growth that relate to resource access, principally food and refuge. This study aimed to identify and characterize behaviours and to make a preliminary investigation of their occurrence under tank culture. Juvenile *Jasus edwardsii* were examined in a flow-through seawater system using a remote video camera system. Twenty-nine behaviours were divided into three sub-groups: aggressive (11), avoidance (6) and others (12). Aggressive behaviours included attacks, pushing, lifting, clasping and carrying an opponent. Avoidance behaviours included moving away in a backwards-, forwards- or side-stepping motion as well as with more vigorous tail flips. These behaviours were components of twelve behavioural groups that described contact, attack and displacement between individuals. Activity was crepuscular with two clear peaks, one in the morning and the other in the evening. The occurrence of behavioural groups was not different between the morning and evening. The frequency of aggressive behaviours was not affected by changes made to stocking density or access to food. The implications of agonistic behaviours are discussed further in relation to developing aquaculture.

## Introduction

Spiny lobster species are found around the world from tropical to temperate seas, they are important in relation to conservation, fisheries and aquaculture (Green et al. 2010). The Southern rock lobster, *Jasus edwardsii*, is a temperate species with established and well managed fisheries in Australia and New Zealand (Holland et al. 2005, Linnane et al. 2012, Linnane et al. 2013). It too has also been under consideration as a species with aquaculture potential (McKoy 1979, Crear et al. 2000, Simon and James 2007). Some advantages relate to reported behavioural characteristics that make it amenable to group culture and arguably more suitable for aquaculture than other lobster species, particularly clawed lobsters (McKoy 1979, Huber and Kravitz 1995, Thomas et al. 2003).

Clawed lobsters exhibit an array of aggressive behaviours, they readily fight to establish and maintain dominance hierarchies and therefore exhibit behaviours that are not ideal for high productivity under typical intensive communal aquaculture systems (Karavanich and Atema 1998, Kravitz 2000, Gherardi et al. 2010). Agonistic behaviour has important consequences under confined aquaculture conditions that relate to access to resources, principally food and refuge, and that encompass direct effects, such as damage or death of protagonists, and indirect effects on growth and health (Thorpe and Huntingford 1992, Drengstig and Bergheim 2013). Hierarchical social structures often lead to feeding hierarchies where there is unequal distribution of food between individuals, differences in food intake result in growth depensation (McCarthy et al. 1992, Winberg et al. 1993). Growth depensation is an increase in variance of a size distribution with time due to individuals growing at different rates (Magnuson 1962, Carter et al. 1996), and its management is critical for aquaculture production (Thorpe and Huntingford 1992, Drengstig and Bergheim 2013).

Compared to clawed lobsters (Huber and Kravitz 1995, Drengstig and Bergheim 2013) less is known about the agonistic behaviour of spiny lobsters, especially in relation to aquaculture (Thomas et al. 2003, Moyle et al. 2009). Spiny lobsters exhibit an extensive range of behaviours including agnostic behaviours which are particularly prevalent around obtaining and retaining shelter (Fielder 1965, Berrill 1976, Cobb 1981, Segura-García et al. 2004). Spiny lobsters aggregate in and around shelters in the wild, yet they also compete for shelters and show aggression around them (Childress 2007). The more solitary species of spiny lobsters are considered more aggressive (Childress 2007) and *Jasus edwardsii*, the subject of the current study, appears more gregarious and less aggressive than some other spiny lobsters (Fielder 1965, Berrill 1976, James et al. 2001, Moyle et al. 2009). Chemicals in the urine and physical contact are the important mechanisms mediating social interactions including aggregation, mating, and agonistic behaviours in spiny lobsters (Briones-Fourzan et al. 2008, Horner et al. 2008, Shabani et al. 2009). Agonistic interactions often involve overt aggressive and submissive behaviours, published information on the nature of these interactions in captivity is spread across spiny lobster taxa *Jasus* (Fielder 1965, Thomas et al. 2003) and *Panulirus* (Berrill 1976, Cobb 1981, Segura-García et al. 2004, Moyle et al. 2009). Consequently, the current study aimed to identify and characterize behaviours of *Jasus edwardsii* and to make a preliminary investigation of their occurrence under tank culture conditions and in relation to some key factors that can be manipulated in order to improve lobster growth and productivity.

## Methods

Juvenile *Jasus edwardsii* stock were caught as puerulus from the East coast of Tasmania and maintained in 4 m^3^ rathbun tanks. Stock and experimental animals were held at 18 °C, and fed on a mixed diet of whole blue mussel, frozen squid and a commercial prawn feed. Lobsters held under standard conditions were used in experiments (Crear et al. 2000). Only the prawn feed was used during the experiments and fed at 1% body weight per day (Thomas et al. 2003). All animals survived and were returned to stock, they were only used once.

Behaviours were examined in two rectangular 25L (width 300 × length 500 × depth 250 mm) tanks that were part of a flow-through seawater system. The flow rate was 7.4 L min^-1^ so that water was replaced approximately 18 times per hour. The system was enclosed by black plastic sheeting to control photoperiod and minimise visual disturbance from external sources. Light was provided from fluorescent tubes and a 12:12 photoperiod used with lights-on at 06:00 and lights-off at 18:00. An infrared light provided illumination for the camera during the dark phase. Each tank system was equipped with a video camera and the tanks were stocked with 5 animals (equivalent to 33.5 individuals per square metre, ind. m^-2^). The carapace was marked to identify each individual. The floor of each tank was marked with a 100 × 100 mm grid so distance and direction of movements could be assessed more easily.

An initial assessment of activity was made on two groups of five animals that were monitored continuously for seven days. Focal sampling (Altmann 1974, Shelverton and Carter 1998) was used to record activity for the first ten minutes of every hour. The unit of activity was defined as movement equivalent to half the length of the lobster. Peaks of activity occurred in the morning between 07:00 and 10:00 and in the evening between 18:00 and 21:00. Twenty-nine component behaviours were identified and used to define twelve behavioural groups (see Results) including when no behaviour was observed (none).

Four new groups of five animals were used to investigate the occurrence of the behavioural groups. Occurrence was the number of times a behavioural group was observed as the first behaviour exhibited during each period of observation: each occurrence of behavioural unit was given a score of 1, observations were made over five days and data adjusted to one hour of observation (Moyle et al. 2009). The effect of time of day was then analysed using these four groups to compare occurrence of behavioural groups between the morning (AM) and evening (PM) periods. The effects of stocking density and feed availability on the occurrence of behavioural groups were measured in separate experiments. The experiments on stocking density and feeding availability were of a preliminary nature and each used 2 groups (10 animals). Stocking density compared standard with a higher density: density was doubled from the standard density of 33.5 to 67.0 ind. m^-2^ by dividing the tanks in half (one half remained empty during the experiment). The effect of feed availability was investigated by comparing one with four feeding stations: the standard single feeding station in the centre of the tank or using four feeding stations simultaneously, one in each corner of the tank (the total ration was the same).

For each experiment the same sampling regime was used: eight 15 min time blocks, four from the morning (05:00–06:45) and four from the evening (18:00–19:45) periods of peak activity, were analysed and behavioural group recorded against individuals (occ. h^-1^). Mean group occurrence was calculated for each behavioural group as total occurrence divided by the number of individuals (five) and standardised to an hour of observation, it was expressed as occurrence per individual per hour (occ. Ind^-1^ h^-1^). Non-parametric statistical analysis using the Kruskal-Wallis test followed by multiple comparison using all pairwise comparisons was conducted due to preliminary nature of the data and the lack of homogeneous variance according to Levene’s test. Statistical analysis was conducted using SPSS Statistics version 22.

## Results

### Component behaviours

Twenty-nine component behaviours were identified and described; 11 aggressive and 6 avoidance (Table 1) as well as 12 others (Table 2). A selection of line drawings to represent aggressive (Figs 1–4), avoidance (Figs 5–8) and other (Figs 9–12) behaviours are presented.

**Figures 1–4.**
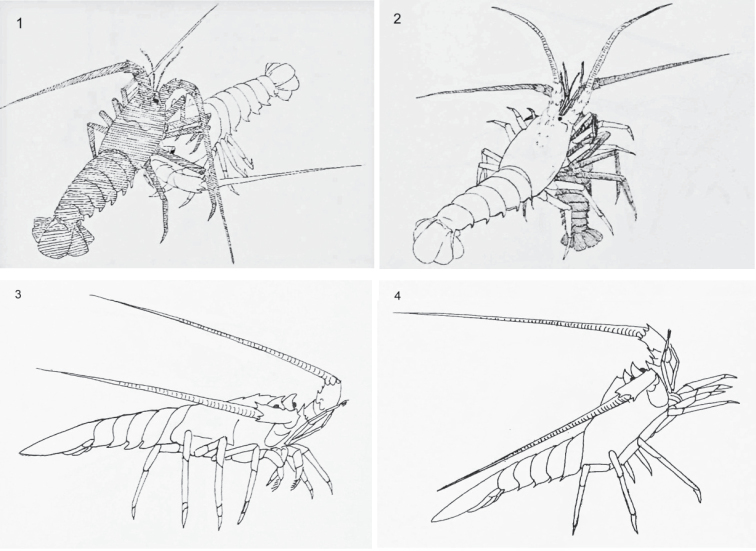
Selected aggressive behaviours of *Jasus edwardsii*: **1** attack 1 (low intensity) **2** clasp **3** rise 1 (low intensity) **4** rise 3 (high intensity).

**Figures 5–8.**
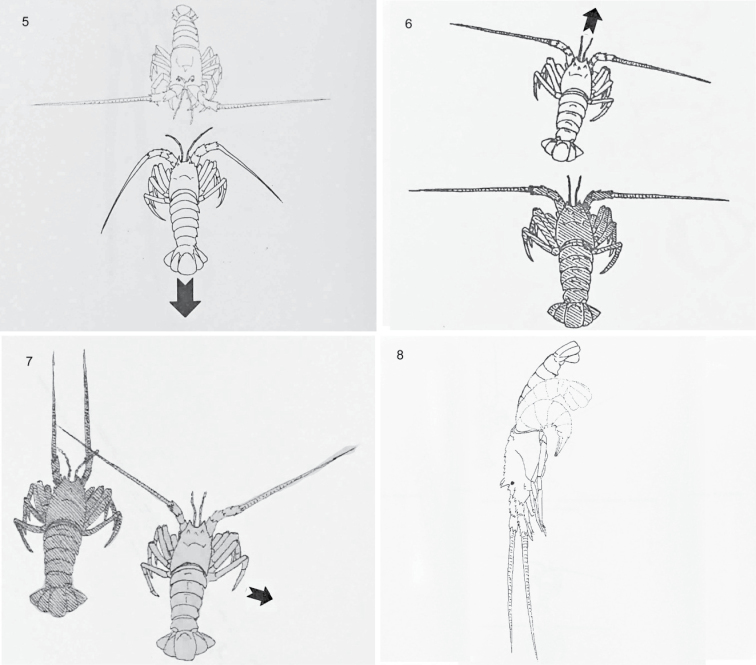
Selected avoidance behaviours of *Jasus edwardsii*: **5** back away **6** walk away **7** side step away **8** flee.

**Figures 9–12.**
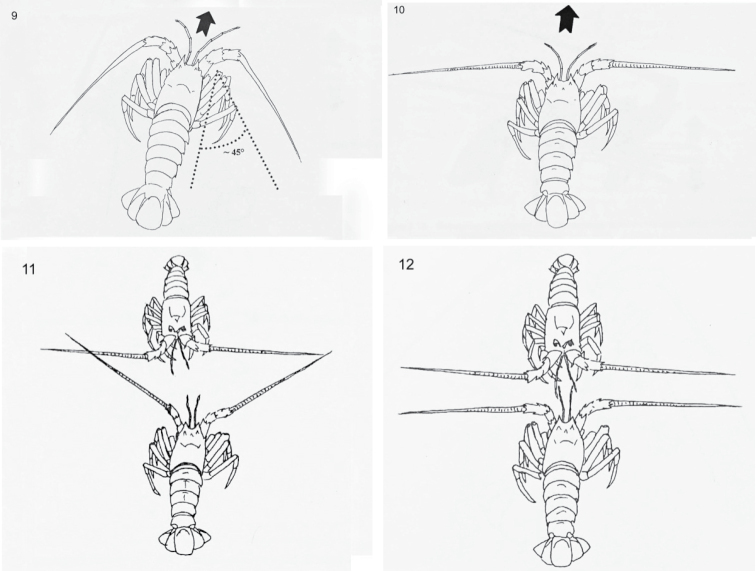
Selected other behaviours of *Jasus edwardsii*: **9** cautious approach, **10** approach forward **11** anntennae touch **12** antennule touch.

**Table 1.** Component behaviours of *Jasus edwardsii* grouped as aggressive or avoidence behaviour.

Behaviour	Description
**Aggressive**	
Attack (A1)	Moderate contact with opponent (see Fig. 1).
Attack (A2)	Vigorous contact with opponent accompanied by clasp (CL).
Burrow (BU)	Vigorous contact lifting opponent clear of substrate.
Carry (CA)	Transport of opponent.
Chase (CH)	Vigorous pursuit of opponent.
Clasp (CL)	Clasp opponent with first three walking legs (see Fig. 2).
Dislodge (D)	Moderate contact in which aggressor dislodges opponent from position.
Push (P)	Vigorous contact in which aggressor displaces and pushes opponent away from position.
Rise (R1)	Orientate toward opponent and raise anterior by extending walking legs (see Fig. 3).
Rise (R2)	Orientate toward opponent and raise anterior above posterior by extending third, forth and fifth walking legs.
Rise (R3)	Orientate toward opponent and raise anterior high above posterior by extending third, forth and fifth walking legs. Accompanied by waving first two pairs of walking legs at opponent (see Fig. 4).
**Avoidance**	
Back away (BA)	Moderate backwards movement away from opponent with tail undulation (Fig. 5).
Flee (FL)	Vigorous movement away from opponent propelled by tail flapping (see Fig. 8).
Move away (MA)	Moderate movement away from opponent with no further interaction.
Short distance escape (SDE)	Vigorous movement that separates opponents.
Sidestep away (SSA)	Moderate sideways movement away from opponent (see Fig. 7).
Walk away (WA)	Moderate movement away from opponent (see Fig. 6).

**Table 2.** Component behaviours of *Jasus edwardsii* other than aggressive or avoidence behaviour.

Behaviour	Description
Antennal pointing (AN)	Orientation of antennae towards approaching opponent whilst first pair of walking legs raised above substrate.
Antennal touch (AT)	Contact with antennae (see Fig. 11).
Antennule touch (AT2)	Contact with antennules (see Fig. 12).
Approach backwards (APB)	Moderate tail-first movement towards opponent.
Approach forwards (APF)	Moderate head-first movement towards opponent with antennae at 90° to body.
Body touch (BT)	Contact opponent with body, usually aimed at dorsal carapace or head.
Cautious approach (CA)	Slow head-first movement towards opponent (see Fig. 9).
Depress (DE)	Body is flattened on substrate and legs drawn tightly into the carapace.
Face-to-face (FTF)	Head to head orientation, anntennae touching and usually involves contact with antennules.
Quiescence (Q)	Stationary with movement of second and third walking legs to ventillate gills.
Side touch (ST)	Moderate sideways movement and contact with opponent.
Toward (U)	Moderate movement towards opponent (not APB or APF).

### Behavioural groups

Twelve behavioural groups, including one for no behaviour, were defined. Behavioural groups were divided into four broad categories (Table 3). Low Intensity Contact (LIC) involved brief contact via mutual antennal touching before separation. Medium Intensity Contact (MIC) was the same as LIC with additional contact where the legs of one animal came into contact with the dorsal surface of the other. Overtly aggressive behaviours were divided between Attack and Displacement. During an aggressive interaction the aggressor and the subordinate exhibited different component behaviours (Table 3).

**Table 3.** Behavioural groups of *Jasus edwardsii* under main categories: low intensity contact, medium intensity contact, attack and displacement. Subordinate behaviour during attack is in parentheses.

Behavioural Group	Behaviours
**Low intensity contact (LIC)**	
LIC1	Approach forward, antennal touch, walk away.
LIC2	Cautious approach, antennal touch, walk away.
**Medium intensity contact (MIC)**	
MIC1	Approach forward, antennal touch, body touch, walk away.
MIC2	Cautious approach, antennal touch, body touch, walk away.
MIC3	Approach forward, antennal touch, side touch, walk away.
MIC4	Cautious approach, antennal touch, side touch, walk away.
**Attack**	
LIA, Low intensity attack	Cautious approach, rise 2, antennal touch, attack 1, (flee), walk away.
MIA, Medium intensity attack	Cautious approach, antennal touch, (back away), chase, rise 2, antennal touch, attack 2, clasp, (flee).
HIA, High intensity attack	Approach forward, rise 3, attack 2, clasp, (short distance escape), chase, rise 3, clasp, (short distance escape), (flee).
**Displacement**	
FD, Forced displacement	Burrow, carry, dislodge, walk away.
D, Dislodge	Approach forward, side touch, push, walk away.

**Figure 13.**
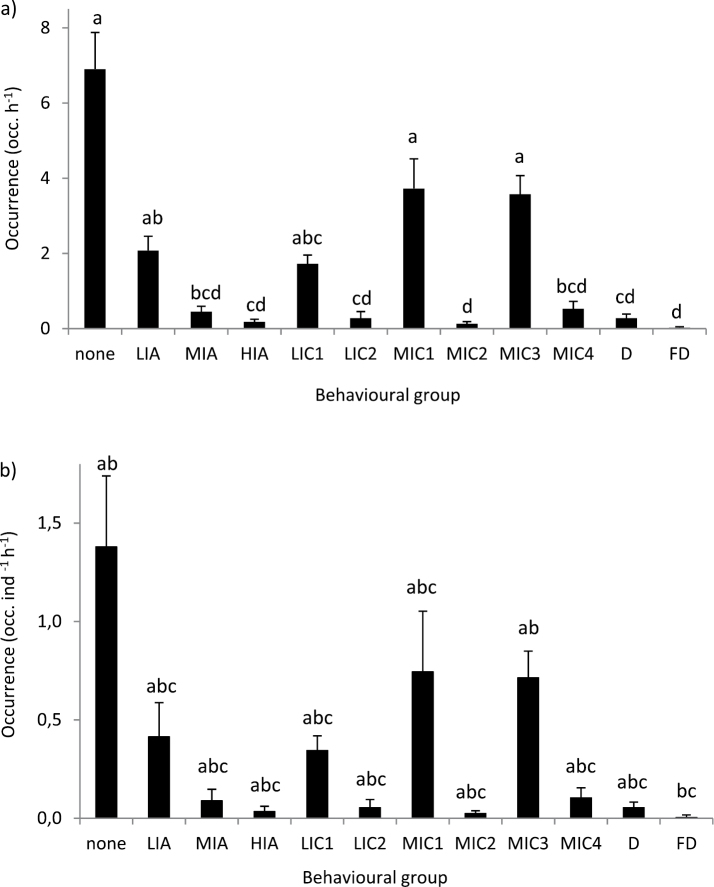
Occurrence of *Jasus edwardsii* behavioural groups as a) mean (± SE) individual occurrence (occ. h^-1^) and b) mean (± SE) group occurrence per individual per hour (occ. ind^-1^ h^-1^). Individual occurrence (a): significant difference amongst behavioural groups (N = 20, P < 0.001, Kruskal-Wallis Test). Group occurrence (b): significant difference amongst behavioural groups (n = 4, P < 0.001, Kruskal-Wallis Test). No significant different amongst means with same letter.

There were statistically significant difference in the occurrence of behavioural groups (Fig. 13). The highest individual occurrence of behavioural groups was for no behaviour, followed by medium intensity MIC1 and MIC3 and by low intensity LIA and LIC1. There were no statistical differences amongst these behavioural groups (Fig. 13a). The same pattern was also apparent when mean group data were analysed (Fig. 13b). These data considered the number of types of behavioural groups averaged across all five individuals in a tank. Other than exhibiting no behaviour, the predominant component behaviours were approach forward (Fig. 10), antennal touch (Fig. 11), side or body touch and walk away (Fig. 6). The occurrence of LIA also included rise 2, attack 1 (Fig. 1) by the aggressor and flee (Fig. 8) by the recipient (Table 3). Conversely, behaviours unique to medium and high intensity attack, such as chasing and clasping, as well as forced displacement and dislodge were rarely observed. There were no significant differences between morning and evening (Kruskal-Wallis test, n = 4, d.f. = 1, P = 0.744), standard and higher stocking density (Kruskal-Wallis test, n = 4, d.f. = 1, P = 0.616) or using four rather than one feeding station (Kruskal-Wallis test, n = 4, d.f. = 1, P = 0.724). Thus, there was no evidence that changes made here to the standard density and feeding management had any influence on behaviours. The pattern of behavioural groups was generally similar to that presented above with LIA, LIC1, MIC1 and MIC3 having the highest occurrence. However, replication was insufficient for further analysis.

## Discussion

Agonistic behaviour has been divided into approach- and avoidance-orientated behaviour (Huber and Kravitz 1995). Clawed lobsters exhibit extensive agonistic behaviours, they are aggressive, solitary and cannibalistic when cultured together (Drengstig and Bergheim 2013). The American lobster, *Homarus americanus*, has an extensive repertoire of aggressive behaviours that include use of the large claws to signal, touch, manipulate and damage opponents. An important aspect of aggressive bouts is to displace opponents, this includes attempting to lift and turn them on their backs as well as pushing or pulling them away from the substrate (Huber and Kravitz 1995). In contrast, many spiny lobsters are reported to be gregarious and only vulnerable to cannibalism following moulting and might, therefore, be predicted to exhibit behaviours that facilitate co-habitation (Childress 2007). However, in wild *Jasus lalandei* this was a function of the capacity of the shelter and smaller individuals were actively displaced by larger individuals (Fielder 1965). Furthermore, in aquaculture the local tank environment is a critical factor that influences behaviour with consequences on growth and production (Thomas et al. 2003, Moyle et al. 2009). The current research aimed to make a detailed study of *Jasus edwardsii* behaviours and behavioural groups in order to provide a basis for understanding the effects of the aquaculture environment.

### Behaviours

Thirty-two behaviours were observed in the spiny lobster *Panulirus cygnus* under a variety of situations in the wild or during captivity and sixteen related to aggressive interactions including five linked to subordinate responses (Cobb 1981). In bouts between pairs of clawed lobster, *Homarus americanus*, seventeen behavioural components were recognised (Huber and Kravitz 1995). “Approach” and “lunge” described slow and rapid approach behaviour, respectively, and corresponded to cautious and forward approach used in the current study. Moderate avoidance behaviour “retreat” was represented in more detail in the current study by move away, back away, walk away and sidestep away whereas the more vigorous “tailflip” matched flee. Avoidance behaviours exhibited by *Jasus edwardsii* were also characteristic of *Panulirus cygnus* (Cobb, 1981). In contrast to *Homarus americanus*, *Jasus edwardsii* exhibited several forms of contact, termed touch, between individuals that were part of low and medium intensity contact behavioural groups and that were not obviously agonistic. When alone *Panulirus cygnus* would sometimes straighten and hold their legs horizontally and suddenly settle in a behaviour termed splay (Cobb 1981), this was somewhat similar to “depress” in *Jasus edwardsii* except the legs were drawn in. All these species orientated towards an aggressor or on-coming animal and raised their anterior body up. The large claws of *Homarus americanus* are an obvious difference between the two types of lobster and were used extensively in combat and seven behaviours identified. *Jasus edwardsii* exhibited different aggressive behaviours that involved displacement through pushing, burrowing and carrying as well as clasping the opponent. The long antennae are used aggressively and defensively by spiny lobsters (Fielder 1965, Cobb 1981), in the present study all eight low and medium intensity behavioural groups included antennal touch as a component behaviour. Interestingly, the high intensity attack did not involve antennal touch and escalated rapidly to attack. This suggested that the presence of chemical signals from urine may have played an important part in communicating social status. In *Panulirus argus* urine is important in reducing the levels of aggression in formed hierarchies; dominant animals increase urine release when engaged in interactions while subordinate animals do not (Shabani et al. 2009). The development of effective hatchery rearing of lobsters, whether on-grown or raised entirely in the hatchery, presents an opportunity for re-stocking. One relevant issue is the behaviour of hatchery raised lobsters to predators (Oliver et al. 2006, Mislan and Babcock 2008), the current research did not address this but it is clearly of importance if animals are to be released into the wild. Research suggests that wild caught post-puerulus that were on-grown juveniles retain a level of plasticity in behaviour and around assessing risks of predation and shifting to nocturnal activity to decrease predation risk (Oliver et al. 2006).

### Stocking density and feed availability

Density has some significant effects on behaviour of spiny lobsters under culture conditions, frequency of occurrence was in the order of 10 time lower for small post-puerulii *Panulirus cygnus* stocked at the lowest density of 30 individuals per m^2^ compared with at 60 to 150 individuals per m^2^ (Moyle et al. 2009). In the current study doubling stocking density did not appear to have any effect on the occurrence of any behavioural groups, including the groups that contained attack behaviours. The standard density used here was similar to 35 individuals per m^2^ and doubling it to 67 was within a range that appears appropriate for tank populations of *Jasus edwardsii* (James et al. 2001). For example, our standard density was similar to that used in a long-term eight month growth experiment on *Jasus edwardsii* (Simon and James 2007) in which survival was over 80% for animals fed good quality diets. This is likely to relate to the gregarious nature of *Jasus edwardsii* which would allow this species to adjust to a higher density without any increase in overt aggression. Although not statistically different, there was a numerical increase in the occurrence of no behaviour (none), this meant that at the higher density the number of animals that did not exhibit any behaviour doubled. Although this possible density-effect requires further investigation, *Jasus edwardsii* may have behavioural mechanisms that will allow them to be held communally at high densities. Excellent survival (>90%) was achieved at up to 200 ind. m^2^ in small *Jasus edwardsii* juveniles (2 g), although weight gain over 118 days increased by as much as 80% when stocking density was decreased from 200 to 50 individuals per m^2^ (James et al. 2001). In the present study, the occurrence of aggressive behaviours was not affected by increasing access to feed. Further study will be required to determine whether *Jasus edwardsii* would attempt to defend a food source but their foraging behaviour in the wild and observations made on feeding behaviour in tanks would suggest this is not normally the case. Food type may also influence the response, with whole or half-shell blue mussels being more defendable than small pellets, as well as having a higher nutritional value than commercial prawn feeds currently fed to *Jasus edwardsii* juveniles (Crear et al. 2000, Simon and James 2007).

### Aquaculture

Pilot aquaculture studies support the conclusion from the current study that agonistic behaviours have a low occurrence in *Jasus edwardsii* (Bryars and Geddes 2005, Simon and James 2007). A significant consequence of agnostic interactions is the maintenance of social hierarchies that can translate into feeding hierarchies, unequal distribution of food and differential growth rates amongst individuals (McCarthy et al. 1993). Growth depensation is the increase in variation in individual size within a group of animals and it provides an informative measure that describes changes in production (Koebele 1985). Simon and James (2007) housed small juvenile lobsters (2.85 g) in specially adapted cages in the sea and fed them either green mussels, moist feed, pelleted dry feed or with no extra feed and relied on any available wild prey and bio-fouling. Over eight months fed lobsters increased in weight by up to 10 times with high survival. There were differences in final weight amongst the feeding regimes; in descending order of, green mussels (×10), pelleted feed (×6), moist feed (×4) and no additional feed (×2). Further analysis showed little change in the weight distribution when fed mussel and pelleted feeds but a doubling of the coefficient of variation with no extra feed. Thus, access to adequate feed appeared to determine the amount of growth depensation and suggested that feeding hierarchies were weak when the amount and nutritive value of a feed were both high. Survival was high and there were no instances of limb damage (C. Simon, personal observation), conspecific attacks being one possible cause of limb damage. In a 12 month holding experiment only 2 out of 52 large 700 g lobsters showed any increase in limb damage that could have been directly attributable to agonistic interactions although tail fan damage was significant (Lorkin et al. 1999). Broadly similar observations were made in later experiment using better feeds and in which more animals moulted (Bryars and Geddes 2005). The long term holding experiments showed that *Jasus edwardsii* can co-exist in the same enclosed space without any extreme consequences, direct or indirect, on growth and survival and provide evidence of the species having the behaviours that facilitate this. Further, even with small groups of five individuals as used in the current study, the high occurrence of no behavioural groups (none) and the absence of contact or attack behavioural groups helps explain why the long-term holding experiments were relatively successful. The main issue is clearly related to feed formulation and nutrition and not communal holding of rock lobsters (Crear et al. 2000, Thomas et al. 2003).

## Conclusion

Twenty-nine behaviours were detected and characterised into three sub-groups in this study: aggressive (11), avoidance (6) and others (12). Among the behaviours recorded, the occurrence of medium and high intensity attacks and displacement events were minimal. This suggested *Jasus edwardsii* juveniles are highly gregarious in culture, even at relatively high stocking densities (67 individuals per m^2^). Further research into characterising the agonistic behaviours of various spiny lobster species with potential for commercial aquaculture is warranted. Better characterisation of agonistic behaviours in culture will assist in testing and improving husbandry management strategies which may include the use of chemical cues, better feed management and shelter designs.
